# Novel Technique of Pneumatic Posterior Capsulorhexis for Treatment and Prevention of Posterior Capsular Opacification

**DOI:** 10.1155/2019/3174709

**Published:** 2019-12-21

**Authors:** Ahmed M. Eid, Shaaban Abd-Elhamid Mehany Elwan, Ahmed M. Sabry, Hossam M. Moharram, Ashraf M. Bakhsh

**Affiliations:** ^1^Ophthalmology Department, Faculty of Medicine, Minia University, 61519 El-Minia, Egypt; ^2^Ophthalmology Department, Security Forces Hospital, Riyadh, Saudi Arabia; ^3^Ophthalmology Department, Faculty of Medicine, Al-Faisal University, Riyadh, Saudi Arabia

## Abstract

**Purpose:**

To evaluate a new technique of posterior capsulorhexis using air support to treat primary posterior capsular opacification (PCO) during cataract extraction surgery or to prevent postoperative PCO.

**Setting:**

(1) Ophthalmology department, Faculty of Medicine, Minia University, 61519, El-Minia, Egypt. (2) Security Forces Hospital, Ophthalmology Department, Riyadh, Kingdom of Saudi Arabia.

**Design:**

Prospective, randomized, consecutive case comparative non controlled study.

**Methods:**

One hundred eyes of 100 patients with a mean age of 63.3 years with dense cataract were enrolled in the study. Fifty of them (group (1)) were with primary PCO (discovered during the operations) and fifty (group (2)) with clear posterior capsule. All of the patients underwent phacoemulsification and posterior capsulorhexis using the air to support the posterior capsule. Then, IOL implantations were done between the anterior and posterior capsular rims. Postoperatively, each patient was evaluated for the following: visual acuity (UCVA and BCVA), manifest refractive spherical equivalent (MRSE), intraocular pressure, intraocular lens (IOL) stability, visual axis opacification, and posterior segment complications as retinal breaks, retinal detachment, or cystoid macular edema (CME).

**Results:**

There were no significant differences in UCVA, BCVA, and MRSE. All cases had a clear visual axis, with stable IOL and normal IOP during the follow-up period without posterior segment complications. The VA improved significantly throughout the follow-up periods in both groups without significant clinical difference.

**Conclusion:**

Pneumatic posterior capsulorhexis is a new effective technique for the treatment of primary PCO in dense cataract and for prevention of postoperative PCO with the good visual outcomes and minimal complications. This trial is registered with NCT04007965.

## 1. Introduction

Posterior capsular opacification (PCO) is one of the common late postoperative complications of phacoemulsification and ECCE rather than its presence in a good number of patients of long-standing and hypermature cataract in developing countries. The treatment of PCO by YAG laser capsulotomy usually leads to the famous annoying symptom (Musca) and carries the risk of IOL damage, elevation, decentration, and tilting [1, 2]. Moreover, it may lead to the posterior segment complications (cystoid macular edema, retinal breaks, and retinal detachment). While, there is no reliable treatment for prevention of PCO, this finding arouses our thinking about this novel technique for treatment and prevention of PCO.

The currently available modalities to prevent postoperative PCO are as follows:

- Some surgical modifications such as hydrodissection, repeated nucleus rotation, and meticulous polishing of the lens epithelial cells (LECs) from the anterior capsular rim and the equator. In 1989, David Apple and his colleagues [3] had demonstrated the value of hydrodissection. In 1992, they emphasized that hydrodissection acts as a barrier to migration of equatorial cells to the posterior capsule and this could reduce PCO [4]. Fine described “cortical cleaving hydrodissection” technique, [5] which was designed to break the equatorial adhesions between lens epithelial cells and the capsule, thus clearance of these cells which are the progenitors of PCO. In 2006, they pointed out to the laboratory and clinical evidence that good hydrodissection, coupled with mechanical “scouring” of LECs from the equator may have a beneficial effect on decreasing PCO incidence [6, 7].
- Changes in the IOL design and materials: e.g., the square edge optic heparin coated and acrylic IOL decreasing the incidence of postoperative PCO than with PMMA IOLs of similar design with several studies demonstrated this concept [8–12].
- Pharmacological strategies either to kill the residual epithelial cells or to prevent their proliferation. Moreover, the ideal agent must be toxic to these cells only without being toxic to the corneal endothelium. Experimentally, few agents have been partially successful without clinical application until now [13, 14].

Improvement in lens materials and design are well documented to decrease the incidence of postoperative PCO [15, 16]. Rotation three times of the hydrodissected nucleus prior to phacoemulsification and a second hydrodissection after nucleus removal improve the results [17]. Bimanual irrigation/aspiration may also help to decrease the incidence of postoperative PCO [18].

### 1.1. Current Treatment Options for PCO

Nd: YAG laser posterior capsulotomy has minor complications such as IOP rise [19, 20], and serious potential complications are reported such as corneal perforation in a patient with systemic scleroderma [21]. Other options for PCO treatment are surgical posterior capsulotomy or capsulectomy, either primary (intraoperative) or secondary.

## 2. Patients and Methods

The study was approved by the local ethical board committee and before the surgical procedure, each patient was adequately educated about the surgery and signed an informed consent in accordance with the Declaration of Helsinki. The patients were chosen from the outpatient clinic and operated at El-Minia University Hospital and Security Forces Hospital, from Jan. 2017 to Jun. 2018. One hundred eyes of 100 patients with dense cataract were included in the study. *The inclusion criteria* were as follows: patient age ranged from 50–73 years with a clear cornea, dense cataract, and without any local or systemic causes for the cataract. *Exclusion criteria* were patients with intraoperative positive pressure, high myopia, and axial length more than 25 mm, corneal dystrophy, retinal disease, previous ocular surgery, active ocular diseases, and glaucoma. The study consisted of fifty eyes (group 1) with PCO and fifty eyes (group 2) with clear posterior capsule (discovered intraoperative).

### 2.1. Preoperative Examination

All cases were subjected to complete ophthalmological examinations including UCVA, BCVA, slit lamp biomicroscopy, tonometry, biometery, dilated fundus examination, and medical history, including any systemic diseases, and the data were recorded echographically.

### 2.2. Surgery

After complete phacoemulsification and irrigation-aspiration of cortical matter, we did posterior capsulorhexis using the air to support the posterior capsule and separate it from the vitreous face using the following unique novel technique.

#### 2.2.1. The Novel Technique

**Figure 1:**
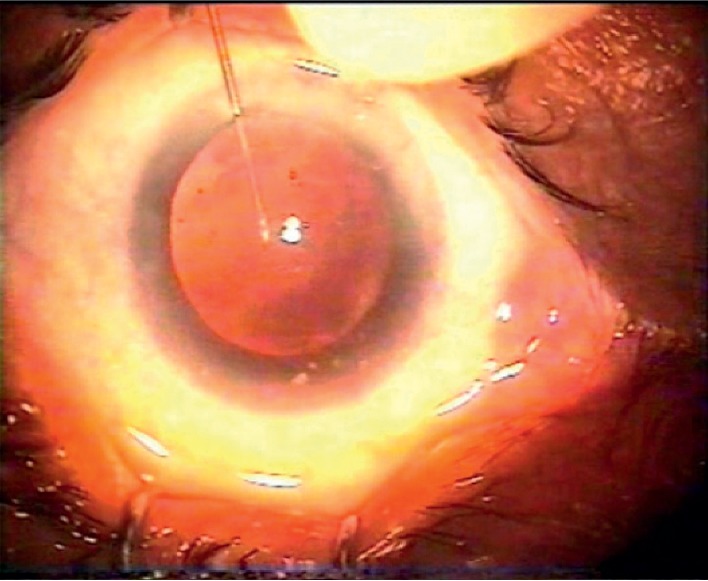
Central puncture of the posterior capsule.

**Figure 2:**
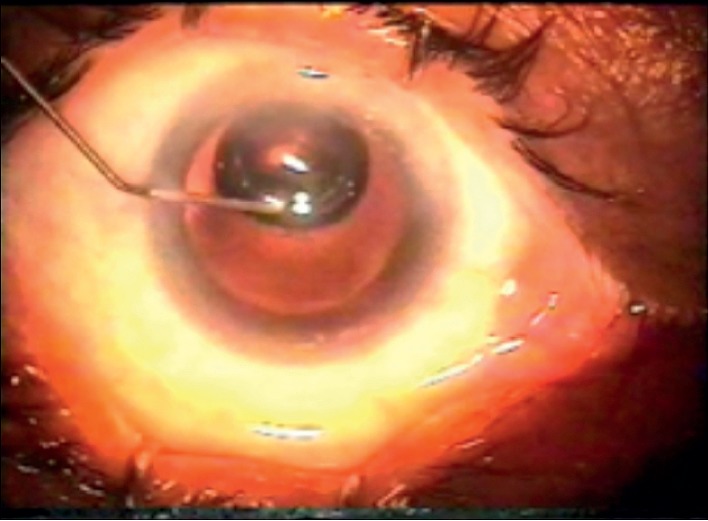
Injection of the air under the posterior capsule.

**Figure 3:**
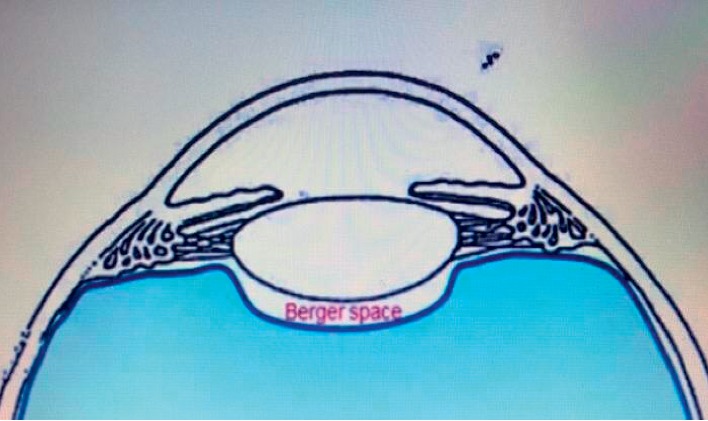
Retrolental Berger space or patellar fossa (animation).

**Figure 4:**
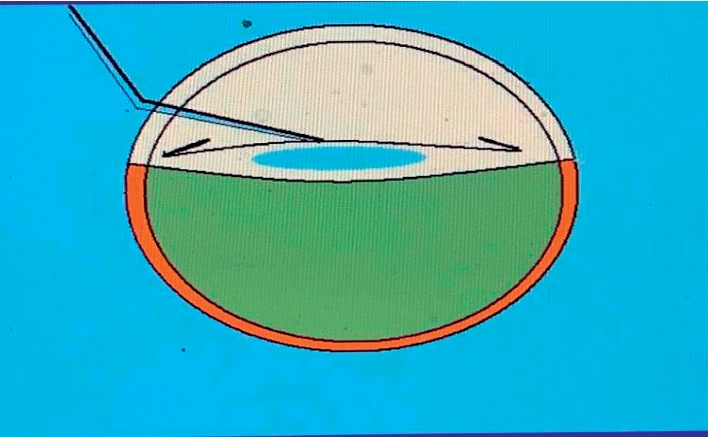
The air elevates, supports, and separates the posterior capsule from the vitreous face (animation).

**Figure 5:**
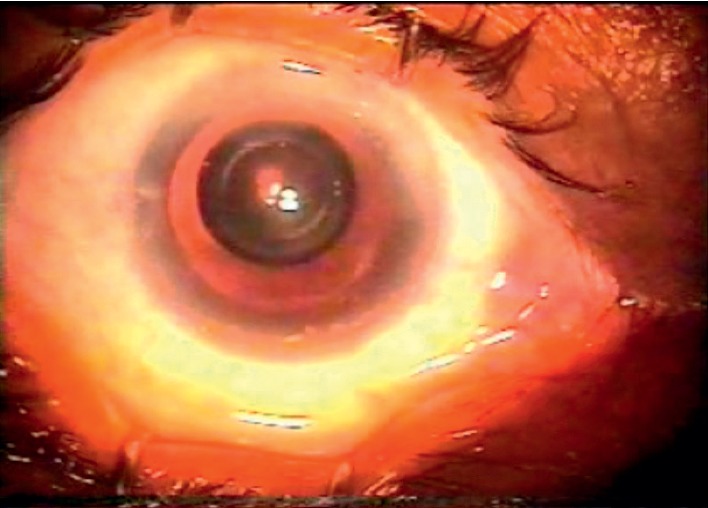
Air under the posterior capsule and viscoat above it.

**Figure 6:**
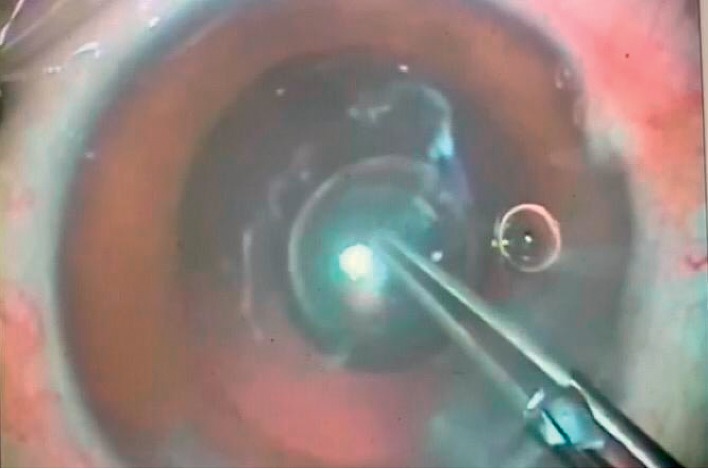
Posterior capsulorhexis using capsulorhexis forceps in a case of primary OPC.

**Figure 7:**
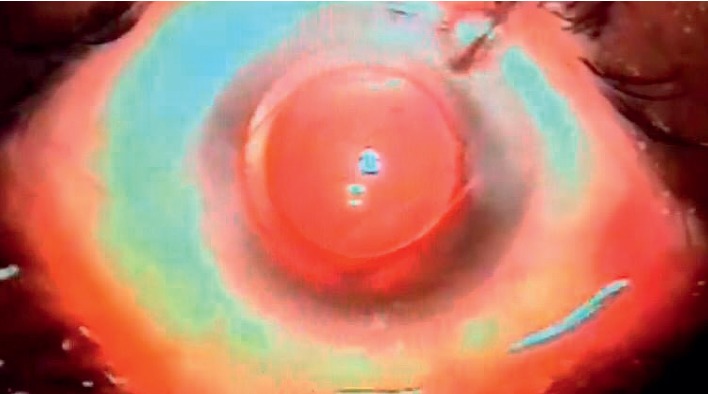
IOL in place between the anterior and posterior capsular rims.

**Figure 8:**
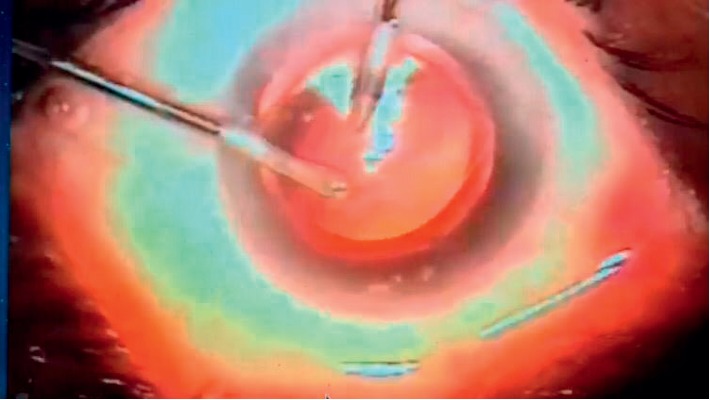
I/A of viscoelastic material.

1. A dispersive viscoelastic material Viscoat (sodium chondroitin sulfate 4%-sodium hyaluronate 3%, Alcon Co) was injected to make the anterior chamber formed without deepening so that the posterior capsule is not forcibly pushed backward and to protect the corneal endothelium.
2. The posterior capsule punctured centrally using cystotome, as shown in (Figure 1).
3. Controllable one-shot injection of 0.1 ml of sterile air through the posterior capsule puncture (Figure 2) into the patellar fossa or Berger space (Figure 3). The air elevates, support, and separates the posterior capsule from the vitreous face (insulin syringe and Healon cannula were used) as shown in (Figure 4).
4. Another viscoelastic injection to the anterior chamber to stretch the posterior capsule and sandwich it between, visco-elastic above and the air below as shown in (Figure 5).
5. Posterior capsulorhexis 4 mm is now performed easily using capsulorhexis forceps (Figure 6).
6. A foldable IOL was implanted between the anterior and posterior capsular rims (Figure 7).
7. Complete the operation with I/A of viscoelastic material (Figure 8) and wound closure followed by eye dressing.

### 2.3. Postoperative Examinations

Postoperatively, each patient was prescribed Tobradex eye drops 0.3% (tobramycin 0.3%—dexamethasone 0.1%, Alcon Co.) with a tapering dose for 1 month and Vigamox eye drops (moxifloxacin 0.5%, Alcon Co.) for 2 weeks. Patients were evaluated at each postoperative visits at 1 day, 1 week, 1 month, 3 months, 6 months, and one year for the following: visual acuity (UCVA and BCVA), MRSE, intraocular pressure, intraocular lens (IOL) stability, visual axis opacification, and posterior segment complications such as retinal breaks, retinal detachment, or cystoid macular edema, and data were registered (Tables 1 and 2).

### 2.4. Statistical Analysis

Patients' data were registered in Microsoft Excel, entered, and analyzed using Sigma Plot-Scientific Data Program for the 2 groups, paired Student's *t*-test was used for the UCVA & BCVA means in decimal values and for MRSE means. For all tests, a *P* value < 0.05 was considered statistically significant.

## 3. Results

The patient's age ranged from 50–73 years, with a mean age of 62.3 years. Fifty males and fifty female patients with dense white cataract were included in the study. Group 1 included fifty eyes with Primary PCO discovered intraoperatively, and group 2 had fifty eyes with clear posterior capsule. There were no intraoperative complications including vitreous prolapse or rupture of the anterior vitreous face. The patients were followed postoperatively for 6 visits one day, one week, one month, three months, six months and one year postoperatively, for MRSE, UCVA, BCVA, IOP, IOL stability, visual axis opacification, and posterior segment complications such as CME, retinal detachment, or retinal break. Tables 1 and 2 show demographic patients' data registration.

The differences were not statistically significant regarding the preoperative mean UCVA and BCVA between the two groups. It was ranging from counting fingers (CF) to hand movement (HM) for UCVA in both groups (*P*=0.99) and for BCVA (*P*=0.95). There were no significant differences at 3, 6, and 12 months in the mean postoperative UCVA comparing the two groups with visual stability (< ±1.25 D difference in two consecutive visits) (*P*=0.82, 1.0 & 0.95): the values were 0.75 ± 0.08 with range, (0.6–0.9), 1.01 ± 0.112 with range, (0.8–1.2), and 1.034 ± 0.085 with range, (1–1.2), respectively, in group 1, while it was 0.80 ± 0.05 with range, (0.7–0.9), 1.03 ± 0.096 with range, (0.9–1.2), and 1.024 ± 0.065 with range, (1–1.2), respectively, in group 2 as shown in (Table 1). The postoperative mean UCVA values were 0.8 in 90% of patients, 1 in 95%, and ≥1 in 100% at 3, 6, and 12 months in group 1, while the corresponding values were 0.85 in 90%, 1 in 96%, and ≥1 in 100% in group 2, respectively. There were no statistically significant differences in the mean postoperative BCVA in both of the groups at 3, 6, and 12 months where the values were 0.96 ± 0.056, 1.036 ± 0.095, and 1.040 ± 0.085 in group 1, and the corresponding values were, 0.993 ± 0.035, 1.037 ± 0.15, and 1.044 ± 0.075 in group 2, respectively, where *P* values were 0.9, 1.0, and 1.0. There were no statistically significant differences between the two groups regarding the mean 1, 3, 6, and 12 months' postoperative manifest refractive spherical equivalent (MRSE) as shown in Table 1; the mean postoperative MRSE was −1.75 ± 2.50 D, −1.25 ± 1.50 D, −0.65 ± 1.35 D, and −0.75 ± 1.05 D in the group 1, while it was −1.65 ± 2.00 D, −1.35 ± 1.25 D, −0.50 ± 1.38 D, and −0.75 ± 1.03 D in group 2, in which *P*  values were (0.88, 0.95, 0.98, and 1.0).

During the follow-up period, no opacification occurs in the pupillary zone and no posterior segment complications reported. In the 2 groups, only one case presented by mild iritis and another three cases with increase IOP in the first visit, which were controlled by intensifying the specific medical treatment. All patients improved completely in the second follow-up visit. All cases in both groups had stable IOL during the follow-up period with significant improvement of the UCVA and BCVA and refraction stability from one visit to the other one (Tables 1 and 2).

## 4. Discussion

Cataract surgery is one of the most common ophthalmic operations worldwide performed. It has been demonstrated to have excellent outcomes, not only in terms of visual acuity and low complications but also in terms of reduced functional impairment and improved quality of life measures [14]. However, posterior capsular opacification (PCO) postoperatively continues to be a challenging problem, and the incidence needs to be reduced to zero [7].

Complete removal of all LECs from the capsular equator by “capsule polishing” techniques is impracticable. Cortical clean-up using “hydrodissection” and “lens fiber stripping” may reduce the incidence of PCO formation by reducing the number of equatorial LECs. Full circumferential capsulorhexis-optic overlap and sharp posterior optic edge did not completely and permanently prevent PCO in all eyes, especially over longer periods. Also, primary posterior capsulorhexis is safe and effective and supplements the efficacy of a sharp-posterior optic edge of IOL forming a “second line of defense”; however, the surgical skill required limits its widespread use.

In Egypt, the incidence of primary PCO is higher because white- and long-standing cataract are more common, and this finding is in agreement with Joshi, [22] in which he reported primary PCO incidence of 38% in longstanding and hypermature cataract. The aim of our study was not only to reduce the postoperative PCO but also to treat the intraoperative primary PCO. While anterior capsulorhexis is easy to perform because of the lens support, posterior capsulorhexis is a little bit difficult because of absence of such a support in which there is a potential, retrolental space (space of Berger), or patellar fossa. Precisely, what we did is we injected the air (0.1 ml) in the patellar fossa through a minute hole in the posterior capsule to cushion, support the posterior capsule, and separate it from the vitreous face and anterior hyaloid membrane during posterior capsulorhexis in cases with primary PCO which may be encountered during phacoemulsification for long-standing cataract and also for clear posterior capsule to prevent postoperative PCO.

In this work, there is a modification of the primary posterior capsulotomy which can be used for the treatment of primary PCO and to prevent postoperative PCO. This technique had reduced the incidence of PCO to zero with minimal complications as postoperative transient rise of IOP. The rise of IOP occurred in two patients out of 50 (4%) in group 1 and in only one patient out of 50 (2%) in group 2 in our cohort and it was controlled by proper medical treatment, and this finding was in agreement with [23] in which they reported a transient rise of IOP after Nd: YAG laser capsulotomy for PCO. By reviewing the literatures, no data could be found about a similar technique to prevent postoperative PCO and to treat the primary PCO. There was some difficulty to implant the IOL between the anterior and posterior capsular rims as it needs meticulous handling and very gentle and slow IOL injection towards the lower part of the equator with good magnification and zooming; we could overcome this difficulty as well as we did the anterior capsulotomy 5.5 mm and the posterior capsulotomy 4 mm. Patients in the study were divided into 2 groups (with or without PCO). As the aim of this study was to manage the primary PCO intraoperatively and to prevent postoperative PCO occurrence in clear posterior capsule in cases of dense cataract with comparing the safety, effectiveness, and results of the novel technique in two different occasions, the primary PCO and the clear posterior capsule because of the posterior capsule behave differently if it is opacified than if it is clear. There was a lack of control group without PCCC as it might be more appropriate; if we do it, we will consider this point on the future study on a larger group of patients, the incidence of PCO is well known in cataract surgery with many literatures, and our study was to evaluate the safety and effectiveness of this novel technique in lowering the incidence of PCO in different capsular types (opacified and clear). By using this technique, we succeeded to decrease the PCO incidence to zero %, and hence there was no need for YAG posterior capsulotomy and its complications.

There are no statistically significant differences in postoperative UCVA, BCVA, and MRSE mean in both of groups as well as during the follow-up period, no opacification occurs in the pupillary zone, no intraoperative complications including vitreous loss or prolapse of anterior vitreous face, and no posterior segment complications reported. All cases in both groups had stable IOL during the follow-up period with significant improvement of the UCVA and BCVA and refraction stability from one visit to the other one. A larger series of patients is needed and is planned to confirm the efficacy and safety of this technique over a longer period of time and follow-up.

## 5. Conclusions

Pneumatic posterior capsulorhexis is a new effective, safe maneuver for the treatment of primary PCO in dense cataract cases and to prevent postoperative PCO with good visual outcomes and fewer complications, and it can be performed in patients susceptible for early postoperative PCO like diabetic and young-age patient.

## Figures and Tables

**Table 1:** Postoperative visual and refractive outcomes in both of the groups.

Parameters	Group 1	Group 2	*P* value
UCVA	Mean	Mean	
3 months	0.75 ± 0.08	0.80 ± 0.050	0.82
6 months	1.01 ± 0.112	1.03 ± 0.96	1.0
12 months	1.034 ± 0.85	1.024 ± 0.65	0.95
BCVA	Mean	Mean	
3 months	0.96 ± 0.056	0.993 ± 0.035	0.90
6 months	1.036 ± 0.095	1.037 ± 0.15	1.0
12 months	1.040 ± 0.085	1.044 ± 0.075	1.0
MRSE	Mean	Mean	
1 month	−1.75 ± 2.50 D	−1.65 ± 2.00 D	0.88
3 months	−1.25 ± 1.50 D	−1.35 ± 1.25 D	0.95
6 months	−0.65 ± 1.25 D	−0.50 ± 1.38 D	0.98
12 months	−0.75 ± 1.05	−0.75 ± 1.03 D	1.0

UCVA = uncorrected visual acuity, BCVA = best corrected visual acuity, and MRSE = manifest refractive spherical equivalent. There were no statistically significant differences in the means of both of the groups. *P* < 0.05 was considered statistically significant.

**Table 2:** Demographics and minimal postoperative complications in both of the groups.

	Group 1	Group 2
Number of patients	50	50
IOL stability	Stable	Stable
Visual axis opacification	None	None
IOP rise	2 cases (4%)	One case (2%)
Iritis	One case (2%)	None
CME	None	None
RD	None	None
Retinal break	None	None

IOP = intraocular pressure, IOL = intraocular lens, CME = cystoid macular edema, and RD = retinal detachment.

## Data Availability

The data used to support the findings of this study are available from the corresponding author upon request.

## Abbreviations

UCVA: Uncorrected visual acuity
BCVA: Best corrected visual acuity
MRSE: Manifest refractive spherical equivalent
PCO: Posterior capsular opacification
CME: Cystoid macular edema
VA: Visual acuity
IOL: Intraocular lens
ECCE: Extracapsular cataract extraction
LECs: Lens epithelial cells
IOP: Intraocular pressure
RD: Retinal detachment
I/A: Irrigation/aspiration.

## Supplementary Materials

Supplementary Materials VIDEO (1). The Steps of the Novel Technique: 1, irrigation/aspiration of cortical matter after finishing phacoemulsification; 2, injection of viscoelastic material; 3, puncture of the posterior capsule with cystitome; 4, air injection under the posterior capsule; 5, pneumatic posterior capsulorhexis; 6, viscoelastic injection and widening of the main incesion; and 7, implantation of the IOL between the anterior and posterior capsular rim. VIDEO (2). Completion of the Novel Technique: 8, irrigation/aspiration of the viscoelastic; 9, IOL in place between the anterior and posterior capsular rim Supplementary File Figure (1): photo of one case of dense cataract from our operated cases. Figure (2): photo of the same case in Figure (1) with primary intraoperative PCO (posterior capsular opacification).
