# Supplementary material for: Novel Technique of Pneumatic Posterior Capsulorhexis for Treatment and Prevention of Posterior Capsular Opacification
Source: J Ophthalmol. 2019 Dec 21;2019:3174709. doi: 10.1155/2019/3174709 (PMC6948290; doi:10.1155/2019/3174709)
Supplement: Supplementary Materials — VIDEO (1). The Steps of the Novel Technique: 1, irrigation/aspiration of cortical matter after finishing phacoemulsification; 2, injection of viscoelastic material; 3, puncture of the posterior capsule with cystitome; 4, air injection under the posterior capsule; 5, pneumatic posterior capsulorhexis; 6, viscoelastic injection and widening of the main incesion; and 7, implantation of the IOL between the anterior and posterior capsular rim. VIDEO (2). Completion of the Novel Technique: 8, irrigation/aspiration of the viscoelastic; 9, IOL in place between the anterior and posterior capsular rim Supplementary File Figure (1): photo of one case of dense cataract from our operated cases. Figure (2): photo of the same case in Figure (1) with primary intraoperative PCO (posterior capsular opacification). [file 3174709.f1.zip › 3174709.f1/NOVEL suppl. file (1).docx]

**
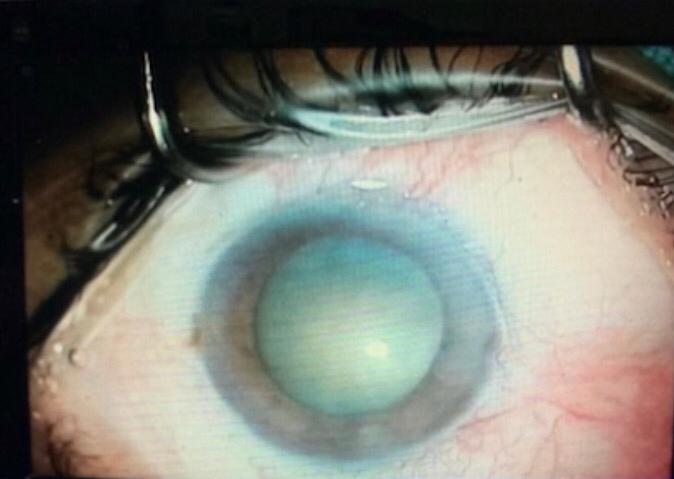
**

**Figure 1. Photo of one case of dense cataract from our operated cases.**


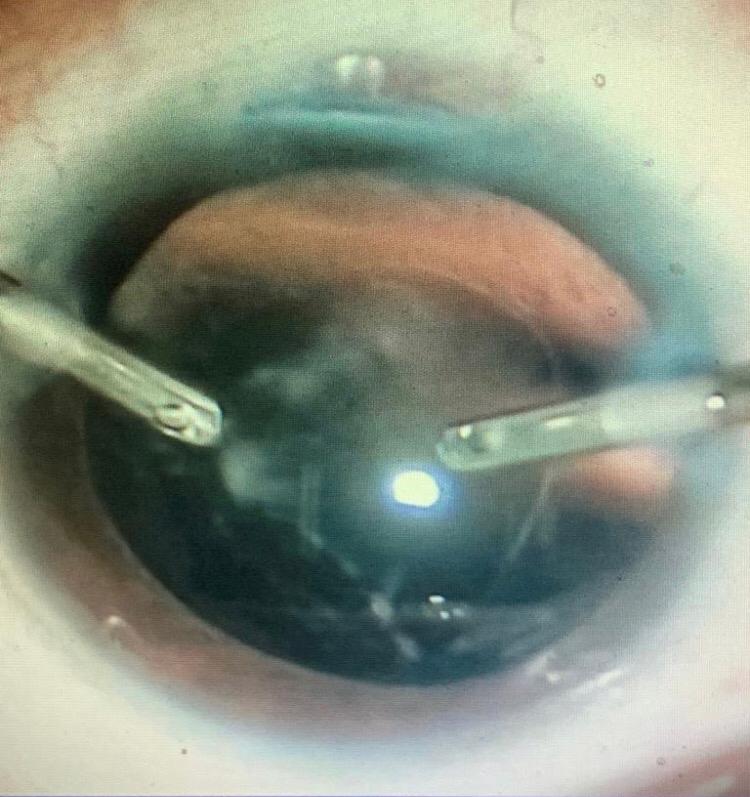


**Figure 2. Photo of the same case in (Figure 1) with intra-operative Primary PCO.**
